# Battey’s Operation

**Published:** 1875-11

**Authors:** D. W. Yandell, Ely McClellan


### Our Exchanges.

---

### BATTEY'S OPERATION.

By D. W. YANDELL, M.D., and ELY McCLELLAN, M.D.

Dr. Battey, of Georgia, having recently done a series of operations in the city of Louisville, several of which we witnessed, we sought an interview with him, and elicited information which we deem of interest to the medical profession. The following is the purport of the interview:

Yandell—What first suggested the idea of this operation to your mind?

Battey—I had charge of a young lady of twenty-one, who had no uterus, but with an active menstrual molimen, whose heart was broken down by the strain upon it in the monthly vascular excitements which were unrelieved, and of which she died. It occurred to me that if I could but divest her of her ovaries the balance would be restored. I searched in vain for a precedent; I dared not to make one.

Yandell—You have performed what you at first called "normal ovariotomy" how many times in Louisville?

Battey—Six times.

Yandell—Have you removed in these cases one or both ovaries?

Battey—From one case one ovary; from another both ovaries at two operations; from three cases both ovaries at one operation.

Yandell—Are you the originator of this operation, or does your procedure differ from that of other ovariotomists so as to make it an original operation?

Battey—I believe the removal of the ovaries with a view to effect the change of life at will is entirely original with myself, both in its conception and execution. I know of no one who desires to claim it for any one else.

Yandell—In what respect does your operation differ from other methods of ovariotomy?

Battey—All other operators propose to remove *ovarian tumors* for the conservation of life and to relieve the patient of an intolerable burden. The vaginal ovariotomy of Thomas and Noeggerath only differs in the *route* by which the tumor is reached. It is my purpose to rid the patient, in the first place, of a *diseased or pernicious ovulation;* secondly, to avail myself of the great alterative changes in the nervous system which attend upon the *change of life;* and in doing so to revolutionize the whole female economy, and thus throw off an *otherwise incurable disease.* It is true that I remove diseased ovaries, frequently cystic; it is true that I sometimes know them to be cystic before removal; but they are ogans which still have sufficient integrity of structure to keep up ovulation, and *it is to stop this diseased or pernicious ovulation that I operate, and to effect the change of life.*

Yandell—Under what circumstances do you think the operation demanded?

Battey—*In the case of any grave disease which is either dangerous to life or destructive to health and happiness, which is incurable by the recognized resources of our art, and which we may reasonably expect to remove by effecting change of life.* I desire it to be distinctly understood that I *do not* propose it for amenorrhœa, nor dysmenorrhœa, nor nymphomania, nor for any other particular malady, but only for such conditions and cases as are alone curable by change of life. *I do not propose it for any case curable by any other method or means.*

Yandell—Will you describe your method of doing the operation?

Battey—I place the patient upon the left side, semi-prone, retract the perinæum with the old-fashioned Sims's duckbill, having a broad but rather short blade that is but little cupped, which I find very desirable. The cervix uteri is now seized with a stout volsella and drawn down under the pubic arch. The incision is made in the median line of the posterior vaginal *cul-de-sac* with scissors, and from an inch and a quarter to an inch and a half in length, the latter preferable. The incision extends at first down to the peritoneum, when, if there be no bleeding, the serous membrane is opened. The speculum is now removed, and the index finger passed into Douglas's fossa to hook down one of the ovaries, and bring it into the vagina, while an assistant, by pressing the hand upon the hypogastrium, depresses the viscera in the pelvis. Occasionally there is advantage in turning the patient supine, that the viscera may gravitate into the pelvis. Often the use of a suitably-constructed forceps is required to assist in bringing down the ovary. A temporary ligature is cast around the base of the ovary, and the other organ similarly treated, when they are removed by *ecrasement*, about ten minutes being consumed in crushing the pedicle of each ovary, the temporary ligatures coming away with the ovaries. No suture is used in the vaginal wound, nor is any tent or drainage-tube employed, as a rule. In several of my cases the ovaries have been found to be bound down by old peritoneal adhesions, which required to be broken up by the finger, and in one case the ovary was literally dug out with the finger-nail.

Yandell—Have you in any way modified or improved your original operation?

Battey—Yes, in two particulars: first, in substituting the vaginal for the abdominal incision; and secondly, in discarding all ligatures and sutures, leaving no foreign body in the tissues. I have not operated through the abdominal wall since my first case. I have used the ligature upon the pedicle in only four cases, and have used the vaginal suture but twice in all my cases.

Yandell—Is the drainage sufficient in all cases?

Battey—The drainage is ample, as a rule. If there be suppuration, I introduce after the first five or seven days a Nelaton soft catheter to wash the cul-de-sac, and prevent premature closure of the wound.

Yandell—Does prolapsion of intestine or omentum through the incision ever occur?

Battey—I have never encountered a case. Usually in twenty-four hours the incision of an inch and a half contracts to half an inch, and in forty-eight hours it is almost entirely closed, barely admitting a catheter.

Yandell—Have you ever encountered troublesome hemorrhage?

Battey—No, never.

Yandell—Should it occur, what means would you use for its control?

Battey—Should I encounter hemorrhage from the incision, I would use torsion to the bleeding vessel; if from an ovarian vessel, I should rely upon ice passed into the *cul-de-sac*, as I did in one of my cases where there was rather free oozing from ruptured adhesions, the patient doing well afterward.

Yandell—Should it become necessary to cleanse the peritoneum, how would you effect it?

Battey—Blood is removed with facility by the finger, if clotted, and drainage of other fluids is adequately effected by turning the patient upon the back. In one case, where I cut through a hematocele to reach the ovaries, I broke up the clot with my finger, and sponged out the cavity with a soft sponge.

Yandell—What instruments do you employ?

Battey—My instruments are few and simple. I have none at all which are made for this operation. Those I use are, *first*, the *old* Sims's speculum before mentioned (I could not operate well with the usual form now employed in gynæcology, but have operated several times with Storer's speculum by reversing the blade); *second*, a stout volsella; *third*, a pair of slender rat-tooth forceps; *fourth*, a pair of long scissors; *fifth*, a pair of old-style bullet-forceps. The operation would be facilitated by suitable instruments, which I have in mind, and hope ere long to have in hand for use.

Yandell—I observe that you use curved scissors to make the incision in the vaginal wall. Would not straight scissors be better?

Battey—I should prefer them straight in the blade, but bent at an angle upon the flat, just back of the joint.

Yandell—How many assistants are required?

Battey—I have operated with three; four are better, and five shorten the time somewhat.

Yandell—How long are you generally in performing the operation?

Battey—Usually an hour. I do not hurry.

Yandell—Do you use ether or chloroform, and have you seen ill effects from either?

Battey—I use only ether, as a rule. Sometimes I employ a little chloroform at the start to overcome the smothering sensation often caused by ether. I have seen no ill effects from either in these cases.

Yandell—How many times have you operated altogether, and with what results?

Battey—Ten times, with eight recoveries and two deaths.

Yandell—What was the cause of death in the two fatal cases?

Battey—In the first fatal case the patient had progressed most favorably to the ninth day, when she was suddenly seized with agonizing abdominal pain immediately upon raising herself up in bed; the pulse ran rapidly up from ninety to one hundred and fifty, and the patient died in twenty-four hours. Autopsy showed a small pelvic abcess, which had contained an ounce of very acrid pus, which had escaped into the peritoneal cavity, where it could find no outlet, the drainage from below having been closed by adhesions. The second death occurred in Louisville, and in the last case I operated on. The patient had for years complained of pain about the heart, and had a very irregular pulse, often running from sixty to one hundred beats in the minute. The cardiac sounds were found by myself and others to be quite normal, and the heart-troubles were believed to be merely functional and sympathetic. She bore the operation well, and it was done with greater ease and facility than in any previous case. On the second day there was a sharp attack of pelvic peritonitis, which extended somewhat to the abdominal peritoneum. On the third day the peritonitis seemed to be subsiding, and the pulse and temperature came down very much. Toward noon the heart showed evident signs of giving way; soon the pulse disappeared at the wrist, while the respiration remained good, the voice strong, and the mind cheerful. A comatose condition came on rapidly in the afternoon, and death occurred on the night of the third day, the action of the heart being extremely feeble, while no pulsation was to be be felt in axillary and femoral arteries for four or five hours before death. Unfortunately an autopsy was peremptorily refused.

Yandell—If any of your other cases have had alarming symptoms, state what they were.

Battey—Of the other eight operations, my first had septicæmia of a threatening character, which rapidly subsided under the peritoneal douche; the fourth had pelvic peritonitis and purulent discharges for a time; and the ninth had pelvic peritonitis and pelvic abcess, which discharged through the vaginal opening.

In five there was no untoward symptom; in three the pulse went at no time over one hundred, and in one it did not at any time exceed ninety.

Yandell—Have the ovaries removed been healthy or diseased?

Battey—In my first case the ovaries were supposed at the time of removal to be healthy, and I am still of this opinion; but they were not carefully examined, as one of my assistants, to whom they were entrusted, negligently allowed them to remain in a piece of cloth for two or three days of very hot August weather, when they were so far decomposed as to render any examination very unsatisfactory. In all the other operations the ovaries removed showed unmistakable disease. In the ten operations I have removed eight cystic ovaries, the cysts varying in size from that of an orange down to that of a cherry. The ovaries removed from two cases here are being subjected to microscopic examination. The report I have not yet received.

Yandell—Is your term "normal ovariotomy" a correct one?

Battey—No; I abandoned that term some time ago, but have as yet no satisfactory substitute.

Yandell—For what conditions, in general terms, have you done these operations, and what have been the results?

Battey—I have operated in widely different circumstances. In one case the patient had amenorrhœa, convulsions, recurring hematocele, repeated pelvic abscesses, incipient tuberculosis from pulmonary congestions, etc. Several of the cases passed under the head of ovarian neuralgia; several had intractable dysmenorrhœa with pelvic deposits of old lymph; one had ovarian insanity, etc. All had exhausted the available resources of the art to no useful purpose. *I operate no case that any other respectable medical man proposes to cure.* In most of my cases the full results of the menopause have not yet been developed. This is the work of many months, and sometimes two or three years are necessary to its full and perfect realization. In no case has the patient failed to realize such a degree of relief and benefit following the operation as to amply compensate her for all the pains and dangers incident thereto, to say nothing of the promise of full and ample recovery at the completion of the physiological "change." In two of my cases this *change* has seemed to occur at once in all its completeness; but it is always my expectation that it will occur gradually, and extending through two or even three years to its final completion. In my first case (now three years ago) the restoration to health is eminently satisfactory. It is true that she is not absolutely and perfectly well, but she is fully relieved of the convulsions, the violent periodical congestions, the hematoceles, the pelvic abscesses, etc., for which I operated. I submit to you the question in all sincerity, if I confine myself to cases where life is endangered, or where health and happiness are destroyed—cases which are utterly hopeless of other remedy this side the grave—ought the profession to demand at my hands the restoration of these forlorn invalids to a state of complete and absolute health in every particular? It is usual for the patients to take on fat freely in a few months after the operation. For the results and prospects of my cases in Louisville, I prefer to refer you to your own observation of the patients themselves.

Yandell—In three of the operations I saw you do, there was plainly cystic degeneration of the ovaries. Had these cysts not been removed, would they, in your opinion, have developed into ovarian tumors, and ultimately have required removal through the abdominal wall?

Battey—Yes, I think so. In two cases I have had the opportunity to watch for some months the progressive enlargement of the cysts. In one case I removed a cystic ovary, and had the opportunity of examining the other ovary, which was entirely healthy. In twelve months this also became cystic, and is now as large as a small egg, and will soon require removal.

Yandell—Do you think it good practice to subject a patient to the hazards of the operation, and remove but a single ovary, though the other may appear to be healthy?

Battey—I do not *now* so think. It is true that I have in three instances removed but one ovary; in two of the cases the other ovary required subsequent removal, and in the third case there is now strong reason to apprehend that a second operation may be required. The conditions for which I operate are so grave that I should not esteem the leaving of one ovary advantageous, though it appear to be quite healthy. Besides, our means of diagnosis in these cases are so imperfect, and the maladies so intractable, that, in order as far as may be to insure the cure, I desire to avail myself of the great alterative changes which attend upon the menopause.

McClellan—What has been the effect of the operation upon the menstrual function?

Battey—I have seen nothing like proper menstruation after the operation. I do not care at present to discuss this branch of the subject, as I purpose to consider it fully at a future time.

McClellan—What has been the effect, if any, upon the sexual desire?

Battey—In my married cases, without exception, it has remained wholly unimpaired. In one unmarried lady I am assured that she is "conscious of no change in her feelings in any respect" since the operation.

McClellan—Have you observed the occurrence of any symptoms indicating that any subject of this operation had been *unsexed* by its performance?

Battey—None whatever. In my cases thus far I am of opinion that the patients upon whom I have operated have, without exception, *lost nothing whatever by the operation.* The married women were *all* hopelessly barren; and the single were presumably barren, because married women in similar circumstances, and with similar organic and functional lesions, are incontestibly barren. There is no loss whatever aside from barrenness.

McClellan—Dr. Matthews Duncan, in his address before the obstetric section of the British Medical Association, refers to this operation "as having been justified by the belief that the removal of the ovaries is the annihilation of all or some of the sexual activities," and applies to it the term "spaying." Have you published any statements that would justify such representation, or does it arise from a misconception of the objects for which the operation is proposed?

Battey—Dr. Duncan seems to greatly misconceive both my objects and my results. This is not peculiar, however, to Dr. Duncan; for, I regret to say, I find very few medical writers or medical men who do fully and rightly apprehend me. I hope that the observation of my work and its results by others will soon correct this. The term "spaying," as generally understood in the lower animals, is very inappropriate to the artificial menopause which is effected in women by my operation.

It will be seen from the foregoing that Dr. Battey has performed his operation in this city *six* times in the persons of *five* individuals. Although a sufficient time has not elapsed to develop fully the results which it is hoped are to be obtained, we present the following summary of these cases:

Case I.—Mrs. S., twenty-four years of age, married two years, but has never been pregnant, had suffered severely from dysmenorrhœa, and since her marriage from intense ovarian pain and all the sequelæ of ovarian disease. The menstrual periods were prolonged, and each occurrence added to the intensity of the symptoms. The patient presented extreme emaciation, great nervous prostration, with insomnia and coccyodynia. Operation peformed May, 1875; left ovary alone removed. Dr. Leachman, in whose professional charge this case occurred, states that there has been a decided improvement in the general condition of the patient since the operation. The menstrual function has been regularly performed, attended with but little pain and no prolongation of the period. The patient is urgent in her demands for the removal of the remaining ovary.

Case II.—Mrs. Q., about thirty-five years of age, married for sixteen years, never pregnant, has suffered from dysmenorrhœa and intense ovarian pain throughout her entire menstrual life, also from persistent coccyodynia. Operation performed May, 1875; right ovary only removed. Dr. Edward Richardson, her medical attendant, states that for some weeks subsequent to the operation there was a total subsidence of all pelvic discomfort, with the exception of the coccyodynia. (This case will be again referred to as Case V.)

Case III.—Miss McD., aged twenty-four years, has suffered intensely from dysmenorrhœa during the entire menstrual life; acute ovarian disorder, coccyodynia, vicarious menstruation from bowels, lungs, and skin. Operation performed August, 1875; both ovaries removed. Dr. Leachman, the medical attendant, reports an entire subsidence of all uncomfortable symptoms. The patient has not menstruated since the operation, although the menstrual molimen has been upon one occasion well marked.

Case IV.—Mrs. H., twenty-eight years of age, married twelve years. The first pregnancy occurred a few months after marriage. Had puerperal peritonitis, cellulitis, metritis, all of which became chronic; and during the next few years had several pelvic abscesses, and was left with intense uterine hyperplasia. Within the past three years has been pregnant three times, but in each instance aborted at the fifth or sixth week. Each abortion was followed by acute cellulitis. Each menstruation was followed by an aggravation of all the symptoms. Operation performed September, 1875, in the hope of arresting the menstrual function. A hematocele was evacuated, and both ovaries were removed.

Dr. E. D. Foree, in whose professional care this lady had been for several years, states that as yet sufficient time has not elapsed to determine the results of the operation, the patient having passed through an attack of peritonitis, the formation of a series of pelvic abscesses, and several attacks of malarial fever. She is now considered as convalescent from the operation, and the most favorable results are anticipated.

Case V.—Mrs. Q., before noted as Case II. The previous operation having failed to afford complete relief from the unpleasant pelvic symptoms, in September, 1875, the *left ovary* was removed. The patient recovered from the operation without any unpleasant symptoms. The coccyodynia is, however, persistent.

Case VI.—Miss M., aged twenty-nine years. This case is fully noted in the preceeding remarks of Dr. Battey.

It seems proper that this paper should be closed with an expression of the impressions made upon us by the operations that were witnessed in this city, and this may best be accomplished by a cursory description.

Having been fully etherized, the patient was placed upon the table in the posture of Sims; the perineum was retracted by a speculum; the cervix uteri was grasped with a volcella, and the uterus drawn firmly downward; with scissors an incision was made through the walls of the vaginal *cul-de-sac*, in the line of the fornix vaginæ. The slight hemorrhage which resulted was arrested by the application of cold sponges. The peritoneum was grasped, nicked, and opened to the length of the orignal incision; the forefinger was passed into the *cul-de-sac*; the broad ligaments and fallopian tubes were examined; the position of the ovaries was determined, and one was drawn as closely as possible to the incision, when it was grasped by forceps and drawn through the opening into the vagina for examination. A stout ligature was passed around the gland to serve as a guide in the application of the *ecraseur*, the chain of which was slowly tightened, until after the lapse of ten or twelve minutes the attachments were severed and the gland removed. The same procedure was practiced upon the other ovary; the wound was sponged out, the vagina cleansed, and the patient placed in bed after an almost bloodless operation.

To witness an operation upon a typical case, or to read a description thereof, is to become impressed with its simplicity and the facility with which it is accomplished. Any tyro may perform the initiatory steps, but it requires a profound gynecologist to complete the operation. It demands that the regional anatomy be impressed upon the brain of the operator. It demands an educated finger, by which the least deviation from the normality may be at once determined.

In Case I, performed in the presence of Gross, Sims, and Sayre, the operator hesitated in determining the exact location of the ovary, so altered by disease and surrounded by adhesions was it; and before the final steps of the operation were attempted the experienced diagnostic powers of Sims were called into play. In this case it was found to be impracticable to excise the ovary; it was crushed and scraped out.

In Case II the ovary was found so altered by disease as scarcely to be recognized.

Case III was purely typical of the operation. The ovaries were readily secured and removed in the most brilliant and successful manner.

In Case IV the incision through the vaginal wall opened a considerable hematocele, and in the management of the operation all the nerve and dexterity of the surgeon were demanded, and it is but just to state that the demand was most fully and ably met. In this case the organs were diseased almost beyond recognition, and were firmly bound down by adhesion.

Case V was for the removal of the remaining ovary of the case reported as the second of the series, when all the difficulties of the first operation were met.

Case VI was typical and the counterpart of Case III.

Thus it is seen that in the series of six cases operated upon in this city by Dr. Battey but two were, it might be said, simple operations. The remaining four demanded all the knowledge, nerve, and dexterity of the surgeon to bring them to a successful completion. Of these six cases one was fatal; but that case was not among the number presenting complications, but was the one performed with the greatest ease to the operator, and the one which seemed to promise the best results.

As to the merits of the operation, as to whether it will accomplish all that its bold originator claims, we are unwilling at this time to commit ourselves by any expression of opinion. We fully coincide with Thomas, who writes, "*It is too young as yet to be decided upon, and is unquestionably a procedure which may be greatly abused.*"

As regards this operation a misconception has occurred. *Normal ovariotomy*, not *vaginal* ovariotomy, is claimed as original by Dr. Battey. Upon page 737 of the fourth edition of Thomas is a letter from Dr. Battey detailing his experience in the operation of vaginal ovariotomy, which originated with Thomas; but upon page 723 of the same edition Thomas fully establishes Dr. Battey's claim to precedence *in the extirpation of the ovaries for the immediate accomplishment of the menopause.*

*Normal ovariotomy* has become a misnomer, as demonstrated in the series of Louisville cases. A designation of the operation is demanded by its growth. Sims suggested that it be christened "Batteyize." We look to the author to name his offspring.

It is proposed at a future day to present again the subject of this operation to our readers, but it will be deferred until such time as the results of the operation upon the four ladies now resident in this city may definitely be determined.—*American Practitioner.*

Messrs. Editors: It has occurred to me that the accompanying article, recently communicated by myself to a secular paper, over the signature of "Medicus," might be of some interest to your readers, and if you think so, please publish.

Yours, etc.,

J. P. Logan, M.D.

